# Telomerase Activity is Downregulated Early During Human Brain Development

**DOI:** 10.3390/genes7060027

**Published:** 2016-06-16

**Authors:** Abbas Ishaq, Peter S. Hanson, Christopher M. Morris, Gabriele Saretzki

**Affiliations:** 1 Institute for Cell and Molecular Biosciences, Newcastle University, Newcastle upon Tyne NE1 7RU, UK; A.Ishaq1@newcastle.ac.uk; 2 Newcastle University Institute for Ageing, Campus for Ageing and Vitality, Newcastle University, Newcastle upon Tyne NE1 7RU, UK; 3 Health Protection Research Unit, Medical Toxicology Centre, Newcastle University, Newcastle upon Tyne NE1 7RU, UK; peter.hanson@newcastle.ac.uk (P.S.H.); c.m.morris@newcastle.ac.uk (C.M.M.); 4 Institute of Cellular Medicine, Newcastle University, Newcastle upon Tyne NE1 7RU, UK; 5 Institute of Neurosciences, Newcastle University, Newcastle upon Tyne NE1 7RU, UK

**Keywords:** telomerase activity, brain, hTERT splice-variants, neural stem cells, hTR, expression, development

## Abstract

Changes in hTERT splice variant expression have been proposed to facilitate the decrease of telomerase activity during fetal development in various human tissues. Here, we analyzed the expression of telomerase RNA (hTR), wild type and α-spliced hTERT in developing human fetal brain (post conception weeks, pcw, 6–19) and in young and old cortices using qPCR and correlated it to telomerase activity measured by TRAP assay. Decrease of telomerase activity occurred early during brain development and correlated strongest to decreased hTR expression. The expression of α-spliced hTERT increased between pcw 10 and 19, while that of wild type hTERT remained unchanged. Lack of expression differences between young and old cortices suggests that most changes seem to occur early during human brain development. Using *in vitro* differentiation of neural precursor stem cells (NPSCs) derived at pcw 6 we found a decrease in telomerase activity but no major expression changes in telomerase associated genes. Thus, they do not seem to model the mechanisms for the decrease in telomerase activity in fetal brains. Our results suggest that decreased hTR levels, as well as transient increase in α-spliced hTERT, might both contribute to downregulation of telomerase activity during early human brain development between 6 and 17 pcw.

## 1. Introduction

Telomerase is a ribonucleoprotein comprised of the TERT (telomerase reverse transcriptase) protein and a TR (telomerase RNA) subunit. Its main and best understood function is telomere maintenance. In humans, telomerase activity is high during early fetal development but down regulated soon afterwards in most somatic cell types. However, telomerase is still active in some adult tissues such as many immune cells, endothelium and also inducible in adult stem cells. It is also known that the TERT protein has various telomere-independent functions (for review see [1]). One of them seems to be a protective function of TERT protein in the brain, which could have implications for brain ageing and neurodegenerative diseases [2,3].

In addition to wild type hTERT, various catalytically inactive splice variants of hTERT with splice sites across the reverse transcriptase region have been described [4]. Alternative splicing of hTERT has been found in various human cell lines and tissue types independent of telomerase activity [5,6]. Interestingly, in many cell lines, the majority of cellular hTERT protein seems to consist of catalytically inactive splice variants, specifically the β-splice version which can reach up to 85% of all hTERT RNA [7]. In addition to changes in splicing patterns during development [8,9] and tumorigenesis [10], they can also occur in response to physiological and environmental factors, such as hypoxia [11,12].

While around 21 different hTERT splice forms have been discovered across the 16 exons of hTERT, the best studied are the α and β splice variants [5,6,9,13,14,15,16]. α-spliced hTERT consists of a 36 base in-frame deletion [9,13] and displays a dominant negative function in cells resulting in senescence or cell death due to telomere shortening in various cancer cell lines [5]. β-spliced hTERT modifies splicing at exons 7 and 8 resulting in a 183 base pair deletion (exon skipping), which introduces a premature termination codon resulting in a truncated hTERT protein without RT motifs B to E at the C-terminal domain [9,13,16]. The ratio between wild type and β-spliced TERT was found to determine the level of telomerase activity (TA) in 50 breast cancer cell lines [16]. While the α-splice hTERT version had been shown to suppress telomerase activity in a dominant-negative fashion [5] there is an increasing interest in analyzing whether various hTERT splice forms can exert non-canonical functions. For example, it has been recently demonstrated that the β-spliced TERT protein without any catalytic activity promoted cellular growth and was able to protect from cisplatin-induced apoptosis in breast cancer cells [16]. Likewise, a new splice form lacking catalytic activity was able to stimulate cell proliferation and activate Wnt signaling [6].

HTERT expression does not always correlate with telomerase activity and various non-canonical functions for hTERT have been described recently (see [1] for review). TERT protein has been found to reduce cellular ROS (reactive oxygen species) levels and inhibit endogenous ROS production and apoptosis induction in cultured human cells [17,18,19,20], as well as mouse neurons [3,21,22,23]. Oxidative stress has been shown to trigger nuclear export of hTERT and import into mitochondria [17,18,20,24,25,26].

We have shown recently that hTERT protein persists in the cytoplasm of neurons and activated microglia, but not astrocytes of adult human brain [3]. While TERT protein levels did not change in the hippocampus of control and Alzheimer’s brains at all levels of pathology (Braak Stage I to VI), a greater proportion of TERT co-localized with mitochondria at Braak Stage VI compared to controls [3]. TERT protein was also found to persist in mouse brain where it is thought to exert protective functions [2,23,27,28,29]. Downregulation of TA in the adult human brain could be due to a variety of mechanisms. It has been previously demonstrated that in rat brain, a catalytically inactive, alternatively spliced insertion-containing TERT form predominantly persisted after TA and WT TERT were downregulated [30]. The authors speculated that splice events could have a developmental function in brain development [30]. Thus we were interested in investigating if α-spliced hTERT could be the predominant hTERT splice form in adult human brain. Both the wild type and the α-spliced version would be recognized by anti-hTERT antibodies as used in [3] recognizing the C-terminus, which is conserved in both proteins, while it is lost in the β-splice version. For this reason, we focused on the α-spliced version in our study.

Here, we investigated the regulation of hTR, wild type and α-spliced hTERT expression during human fetal brain development, young and old adult frontal cortices as well as during *in vitro* differentiation of neural precursor stem cells (NPSCs). We demonstrate that TA and hTR both decrease during fetal brain development, while WT hTERT and α-spliced TERT seemed to co-exist in adult human brain. However, while TA was also downregulated during *in vitro* differentiation of NPSCs no changes in the three telomerase-related genes were found suggesting a different mechanism for the decrease in TA compared to human fetal brain development.

## 2. Materials and Methods

### 2.1. Human Brain Tissue

The fetal brains were obtained from the MRC/Wellcome Trust Human Developmental Biology Resource (HDBR), United Kingdom (Ethical permission for the collection and use of this material for research has been obtained at the Institute of Human Genetics, Newcastle and at the Institute of Child Health, London). We used 12 human fetal brains at different stages of development (from pcw 6 until 20) with normal karyotypes (Table 1). Healthy adult brain tissues were from frontal cortices and obtained from the Newcastle Brain Tissue Resource at Newcastle University (NBTR), United Kingdom, after relevant informed consent from donors and in accordance with Newcastle University ethics board and ethical approval awarded by the Joint Ethics Committee of Newcastle and North Tyneside Health Authority (reference 08/H0906/136). All brains were assessed neuropathologically according to published criteria [31,32,33,34] and were free of any disease-related pathologies.

### 2.2. Cells and Cell Lines

SH-SY5Y neuroblastoma cells were obtained from ECACC. HeLa cervix carcinoma cells were obtained from ATCC. Both cell lines were used as positive controls in the TRAP assay and contained equally high amounts of telomerase activity.

### 2.3. Human Neural Precursor Stem Cells (hNPSCs)

The hNPSC line N1997 was isolated from forebrain of a fetus at 6 pcw with the approval from the National Research Ethics System (15/NE/0090). Cells were grown as neurospheres and differentiated, as described in Madgwick *et al.*, 2015 [35]. Briefly, hNPSCs were grown on Geltrex-coated 6-well plates in proliferation medium for 4 days, then growth medium was replaced with differentiation medium, which was changed every other day. Cells were differentiated for 14 days and samples taken at days 0, 2, 6, 10 and 14 of differentiation. Differentiation of hNPSCs produced approximately 70% Tuj-1 expressing neurons and 30% expressing the astrocyte marker GFAP at day 14 [36]. RNA was isolated using TRizol and TRAP lysates obtained with CHAPS buffer (TRAP kit, Roche, Switzerland).

### 2.4. TRAP Assay

Aliquots of the ground fetal brains were lysed with different amounts of lysis buffer depending on the amount of the sample. Samples weighing between 1.7 mg and 4 mg were lysed with 20 μL of CHAPS lysis buffer. Samples weighing between 4 mg and approximately 8 mg were lysed with 30–40 μL of lysis buffer. The rest of the fetal brain samples, and neural stem cell samples were lysed with 50 μL of lysis buffer. Total protein concentration was measured by Bradford assay (Bio-Rad Protein Assay, Hercules, CA, USA) and absorbance at 450 nm was measured using an Omega spectrophotometer (Omega FLUOstar, BMG Labtech, Ortenberg, Germany). Twenty-five microliters of TeloTAGGG PCR reaction buffer and 1 μg of protein to a final volume of 50 μL in RNase free water were used for each sample in the amplification step. Samples were diluted by 1:10 if the required the samples were too concentrated. 100 ng of HeLa lysates was used as a positive control. The TRAP assay was performed according to the manufacturer’s protocol (TeloTAGGG Telomerase PCR ELISA kit, Roche, Switzerland). Absorbance at 450 nm was detected using the Omega spectrophotometer.

### 2.5. Quantitative PCR

RNA isolation was performed using the RNeasy Lipid Tissue kit (Qiagen, Hilden, Germany) according to manufacturer’s protocol. QIAshredder was used for disruption and homogenisation. RNA were eluted in 30 μL of RNase free water. Reverse transcription was performed according to standard protocol. In brief, 1 μg of RNA was added to 1 μL of random primers (Thermo Scientific, Waltham, MA, USA) and made up to 11 μL with RNase free water, then incubated at 75 °C for 7 min. Four microliters of 5× First Strand Buffer (Invitrogen, Carlsbad, CA, USA), 2 µL of DTT (0.1 M) (Invitrogen), 1 µL of RNase Inhibitor (Promega, Madison, WI, USA), 1 µL of dNTP (10 mM stock, each) (NEB, Ipswich, MA, USA) and 1 µL of Superscript Reverse Transcriptase III (Promega) were then added to the reaction mixture. The samples were then heated to 95 °C for 2 min, and incubated at 45 °C for 90 min.

For each primer pair (see Table 2) samples were loaded in triplicates. For the required number of wells on a 0.1 mL MicroAmp Fast 96-well reaction plate (Applied Biosystems, Foster City, CA, USA), 5 μL of SyBr Green (SensiFAST SYBR Hi-ROX, Bioline, London, UK), 3 μL of RNase-free dH_2_O, 0.5 μL of each forward and reverse primer was added to a 0.5 mL microfuge tube. Nine microliters of the mix was then added to each well. One microliter of sample cDNA was added to the appropriate wells. QPCR parameters were as follows: Holding stage at 95 °C for 120 s, cycling stage at 95 °C for 5 s, then 60 °C for 30 s, and melt stage at 95 °C for 15 s, 60 °C for 60 s and 95 °C for 15 s. For hGAPDH and hTR, cycling stage temperature was 60 °C. For α-spliced hTERT and WT hTERT, cycling stage temperature was 53 °C. Conventional PCR was performed with hGAPDH primers using RNA from SH-SY5Y, and 6 pcw samples 1 and 2 to ensure that there was no genomic DNA contamination in the samples.

### 2.6. qPCR Data Processing and Analysis

**Figure 1:**
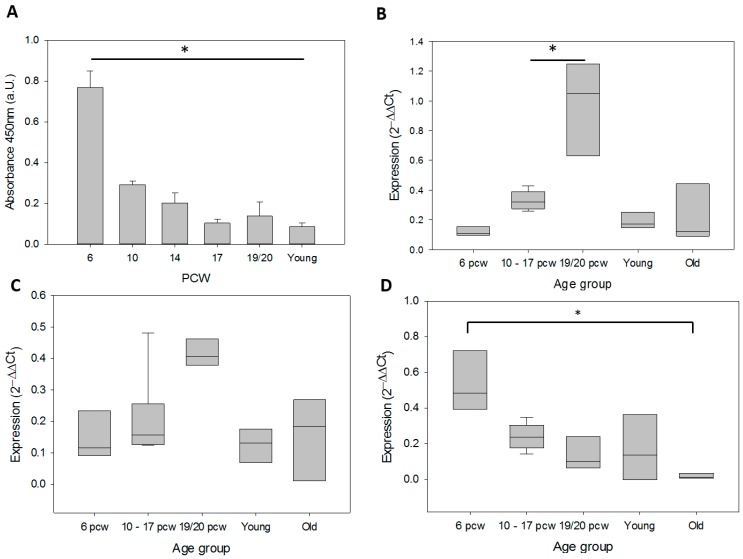
Telomerase activity and expression of telomerase-related genes in fetal brain, young and old cortices. (**A**) Telomerase activity, mean and SD, TA at 6 pcw was significantly different from all other time points; (**B**) α-spliced hTERT, the values for 19/20 pcw are significantly different from those on 10–17 pcw; (**C**) WT hTERT, (**D**) hTR expression at 6 pcw was significantly different from old brains. (B–D) are not normally distributed and have been normalised to SH-SY5Y controls and their 2^−ΔΔCt^ set as 1. The box plots show mean and SD of n = 3 for the 6 pcw and 19/ 20 pcw groups, and n = 6 for the 10–17 pcw groups. Box-plots show the median with 25% and 75% interval in the box, whiskers the 10% and 90%. One way ANOVA (on ranks for (B–D)) and Holm-Sidak *post hoc* tests were performed to determine statistical significance. * *p* < 0.05.

**Figure 2:**
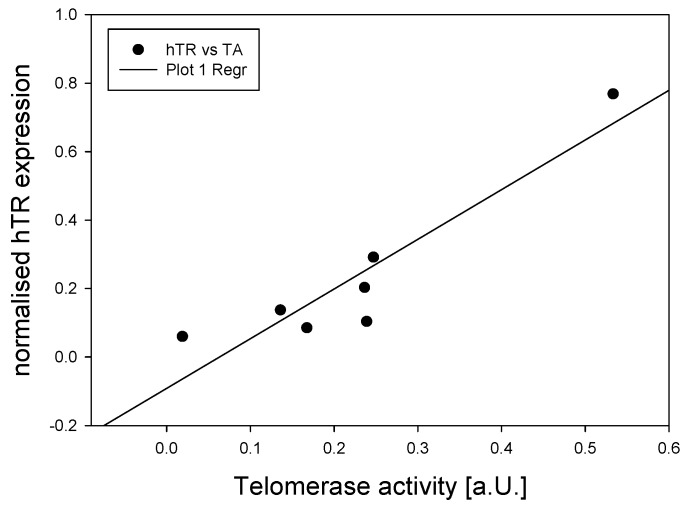
Regression analysis showing a significant correlation between hTR expression and telomerase activity. Data as shown in Figure 1A,D.

**Figure 3:**
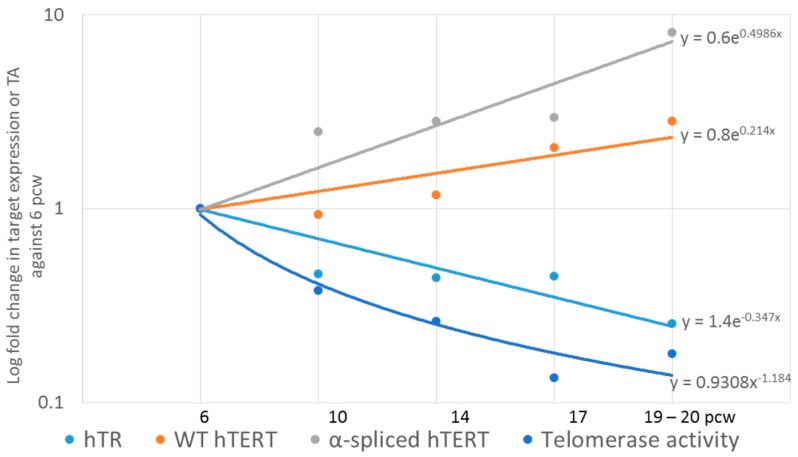
Summary of changes in expression of telomerase-related genes in fetal brain, young and old cortices. Data correspond to those from Figure 1. Telomerase activity and 2^−ΔΔCt^ of all data were acquired by normalizing to 6 pcw instead of SH-SY5Y (in the case of 2^−ΔΔCt^ data), allowing fold change comparison of any time point with 6 pcw. Rate of change in each target over development can be determined from the slope of the graphs.

Gene expression levels were calculated using the comparative Ct method [39]. ΔCt (Ct_target_ − Ct_hGAPDH_) and 2^−ΔΔCt^ (ΔΔCt = ΔCt_sample_ − ΔCt_SH-SY5Y_) were obtained for each technical repeat. This method normalizes the target data to a housekeeping gene first (hGAPDH), then to another sample (SH-SY5Y). In Figure 1 and Figure 2, SH-SY5Y has a 2^−ΔΔCt^ value of 1. The summary of changes in Figure 3 are 2^−ΔΔCt^ where the 6 pcw sample was used for normalizing. Thus, 6 pcw has a value of 1.

### 2.7. Statistical Analysis

**Figure 4:**
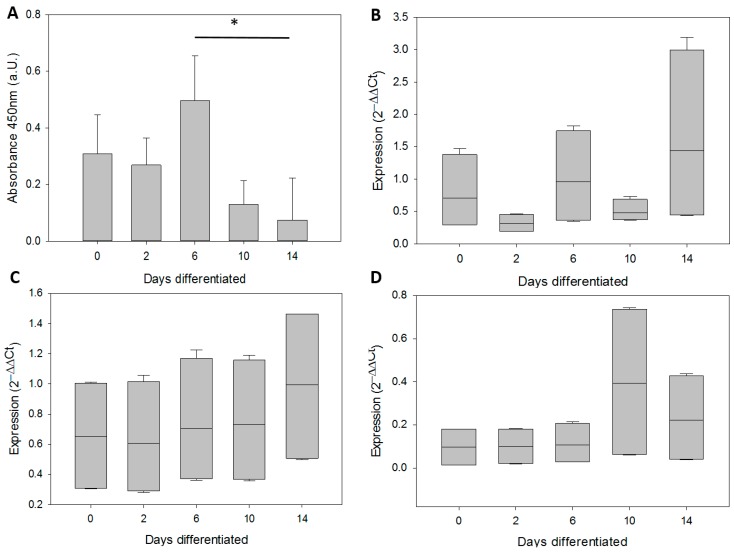
Telomerase activity and expression of telomerase-related genes in neural precursor stem cells and their differentiated progeny. (**A**) Telomerase activity, mean and SD from 2 independent differentiation experiments with significant differences between day 6 and 14; (**B**) α-spliced hTERT; (**C**) WT hTERT; (**D**) h*TR* expression. (**B**–**D**) have been normalised to SH-SY5Y, where 2^−ΔΔCt^ was set to 1. * *p* < 0.05. One way ANOVA (on ranks for (**B**–**D**)) and Holm-Sidak *post hoc* test was performed was performed on these data. The bars show mean and SD of n = 2 (independent qPCR runs) for all groups in (**B**–**D**).

**Figure 5:**
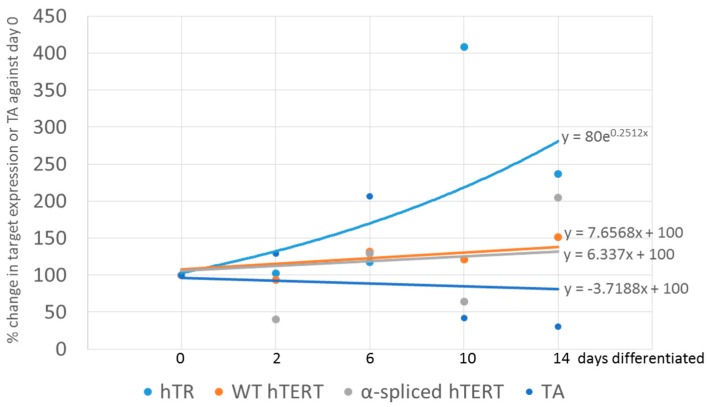
Summary of changes in expression of telomerase-related genes in neural precursor stem cells and their differentiated progeny. Data correspond to those from Figure 4. Telomerase activity and 2^−ΔΔCt^ of all data were acquired by normalizing to 0 days differentiated instead of SH-SY5Y (in the case of 2^−ΔΔCt^ data), allowing fold change comparison of any time point with 0 days differentiated. Rate of change in each target over development can be determined from the slope of the graphs.

All statistics including linear regression analysis was performed and graphs in Figure 1 and Figure 4 created using SigmaPlot 12.5 (Systat Software Inc., San Jose, CA, USA). qPCR data were not normally distributed, and were analyzed by ANOVA on ranks using the post-hoc Holm-Sidak test. Samples from 10 pcw, 14 pcw and 17 pcw were grouped together to increase sample numbers. Slopes for Figure 3 and Figure 5 were generated using Microsoft Excel 2013. Trend lines are shown as either exponential or linear lines were used according to perceived fit of the line to the data points. Y intercept were shifted to ensure that the lines passed through 6 pcw or 0 days differentiated.

## 3. Results

### 3.1. Telomerase During Human Brain Development and in Adult Cortex Tissues

We found a significant (*p* < 0.05) decrease in telomerase activity (TA) for every developmental time point in the fetal brain compared to 6 pcw (Figure 1A). TA was 0.769 ± 0.082 at 6 pcw, then sharply decreased to 0.292 ± 0.019 at 10 pcw, which is still positive, but low. TA below 0.2 is considered to be negative (TeloTAGGG Telomerase PCR ELISA kit handbook), thus, TA in fetal brain tissue seems to be already at negligibly low levels at 10 weeks post-conception. TA in brain samples from young donors was also analyzed and displayed an absorbance unit of 0.085, showing that TA remained negative in adult brains.

α-spliced hTERT expression displayed a trend towards an increase from a mean 2^−ΔΔCt^ value of 0.120 ± 0.018 at 6 pcw to 0.976 ± 0.181 at 19 and 20 pcw, while it increased significantly (*p* < 0.05) between 10–17 pcw and 19 and 20 pcw, from 0.331 ± 0.026 to 0.976 ± 0.031 (Figure 1B). Interestingly, expression then decreased again in adult human cortices, where it was expressed at comparably low levels as in early fetal stage (6 pcw). Thus, there seems to be transient increase in α-spliced TERT which is not maintained into adulthood. However, there was no significant correlation to TA.

WT hTERT expression did not change significantly during both fetal development and in adulthood but showed a trend towards increase similar to the α-spliced hTERT at 19/20 pcw (Figure 1C). Thus, in general, it seems that WT hTERT expression seems rather constant in brain tissue during different ages.

In contrast to the expression of WT and α-spliced TERT, hTR expression followed the general trend of decrease detected for telomerase activity in the analyzed brain tissue (Figure 1D).

Performing a linear regression analysis we found a highly significant correlation between hTR amounts and telomerase activity (Figure 2, *p* = 0.003). Comparing the rates of change in the different parameters measured for brain tissue (Figure 3), α-spliced hTERT amounts increase at a higher rate than the decrease in hTR but did not correlate significantly with the decrease in TA.

### 3.2. In Vitro Differentiation of Neural Precursor Stem Cells (NPSCs)

Since at early stages of brain development there is a major involvement of neural stem cells we determined the same parameters for brain tissues in NPSCs that had been differentiated *in vitro* over the course of 2 weeks. The neural precursor stem cell line N1997 was derived from forebrain at the same stage as the youngest brains (6 pcw). *In vitro* differentiation was performed over 2 weeks and aliquots taken at the indicated days. Telomerase activity showed no significant changes between 0 and 6 days of differentiation (DD) (Figure 4A). In contrast, TA significantly decreased after 6 days and no TA was detected after 10 days of differentiation (*p* < 0.05), corresponding to a differentiated phenotype of NSPC derived cells after 14 days of differentiation, where the cultures would consists of neurons and astrocytes in a ratio of their protein amounts being around 70% from neurons and 30% from astrocytes [36].

Expression of α-spliced hTERT was in general very high and comparable to that of SH-SY5Y neuroblastoma cells (set as 1) throughout the differentiation process (Figure 4B). Although there was a high heterogeneity at 2 weeks, nothing reached statistical significance. WT hTERT transcripts were also comparably high in relation to SH-SY5Y neuroblastoma cells (set as 1) throughout the differentiation process (Figure 4C). Although this result seems expected, as NPSCs from early human embryos should have a high expression of wild type hTERT and TA, it was surprising that it does not correlate to the sharp decrease in TA. After day 6, expression of hTR was also constant at all time points with a very low expression levels at days 0–6, and a slight trend towards an increase at longer differentiation times (Figure 4D). However, none of the expression kinetics determined during differentiation of the NPSCs correlated or could explain the decrease in TA at later days of differentiation. A summary of the changes is provided in Figure 5.

In conclusion, our data show that TA is significantly downregulated during fetal brain development, as well as during *in* *vitro* differentiation of human NSPCs. However, expression levels and kinetics of telomerase related genes did not correspond between both types of experiments. Thus, *in vitro* differentiation experiments of NPSCs do not model the mechanisms of downregulation of telomerase activity in early human brain development.

## 4. Discussion

In this study we analyzed the expression of telomerase activity, as well as the expression of different telomerase associated genes such as α-spliced hTERT, WT hTERT and hTR during fetal brain development and *in vitro* differentiation of neural stem/progenitor cells. Human brain development begins during the third gestational week (GW) with the differentiation of the neural progenitor cells, and lasts into adulthood. By the end of the embryonic period at eight gestational weeks (corresponding to six post conception weeks) the basic brain structures are established and the major compartments defined [40]. During fetal development there is rapid growth and development of both cortical and subcortical structures. Neuron production in humans begins at embryonic week seven, until completion in the middle of gestation (GW 20). After week seven, neurons migrate and begin to form major network structures. At the beginning of neuron production before embryonic week seven, there is a rapid expansion of the neural stem and precursor pool by symmetric division [40]. This is followed by asymmetrical division of NPSCs and cortical neurogenesis is complete in humans by around week 15. From then on neurons migrate in a radial manner from the ventricular zone (VZ) in the center of the brain where the stem cells are located out to the developing neocortex. After reaching their target region in the cortex, the young neurons mature further by forming axons and dendrites in order to form networks and transmit information [40].

A decrease of telomerase activity in fetal human brains from GW 10 (pcw 8) after gestational week 16 has been described previously [8]. We detected high levels of TA at 6 pcw, which corresponds to eight gestational weeks. At this stage there is a high amount of rapidly dividing neuronal stem and progenitor cells present in the developing brain [40,41]. At 10 pcw TA was still detectable, but disappeared between 14 and 17 pcw corresponding well to Ulaner’s results [8] and the onset of the neuronal maturation process [40]. 

The ratio of proliferative NPSCs to differentiated cells (neurons and glial cells) shifts over the course of the development of embryonic and fetal brain with a large amount of symmetrically dividing NPSCs between 4 to 6 pcw [40]. Thus, we suggest that the early developing brain predominantly contains of telomerase positive actively dividing NPSCs. This is consistent with the high TA we observed in brain tissue at 6 pcw. As neurogenesis progresses, progenitors progressively undergo asymmetric division differentiating into mature brain cells. Consequently, the expansion of the progenitor pool gradually slows. [42]. Thus, the decrease in TA during brain development could be attributed to the progressive expansion of the post-mitotic neuronal population and the relative decrease of the NPSC population.

Human astrocytes from old brains, as well as astrocytes in mixed mouse cultures, did not express TERT protein while neurons from the same cultures were positive for TERT protein and positive for telomerase activity at least one week during *in vitro* culture [3], presumably coinciding with a neuronal maturation process.

Importantly, we found that the decrease in TA correlated strongly with a decrease in hTR expression for the corresponding time points. Although hTR expression has been suggested to be ubiquitous in most cell types [43], it has been demonstrated that repression of hTR expression resulted in a rapid growth arrest and a dramatic decrease in TA in MCF7 cancer cells [44]. The authors found that reduction in hTR stimulated ATR activity and elicited a p53/CHK1-dependent cell cycle arrest independent of DNA damage. Others have identified transcriptional repressors of hTR expression such as mdm2 [45]. In addition, in genetic diseases, such as Dyskeratosis congenita, caused by either mutations in the *hTR* gene or defects in dyskerin result in diminished hTR levels that in turn limit telomerase activity and telomere length [46,47].

In contrast, Ulaner and co-authors did not find any changes of hTR expression in fetal heart and kidney tissue using a conventional PCR method until 21 gestational weeks (the last time point fetal tissue can be obtained from clinically indicated abortions) although TA was severely downregulated between post-conception weeks 9 and 13, respectively, in these two tissues (Table 3) [9]. Thus, it seems that hTR had no major contribution in the downregulation of TA in these two tissues. That could mean that different mechanisms are employed for downregulation of TA in different tissue types and the downregulation of hTR as a mechanism to downregulate TA might be more brain specific. Not much is known about hTR expression or its regulation in brain cells (predominantly neurons and astrocytes). Stimulation of TA, hTERT and hTR had been found by the oncofetal protein Pax8 in human glioma [48]. Pax genes play a role in early brain development [49] and their deregulation has been linked cancer types, such as astrocytoma and gliomas.

Correlation of TA with wild type, α-spliced and β-spliced hTERT differs across human fetal heart, liver and kidney development [8,9] (see also Table 3). In fetal kidney, Ulaner and co-authors found that WT hTERT was maintained until 21 gestational weeks (19 pcw) while both TA and WT hTERT were abolished by 15 and 16 weeks, respectively. α-spliced TERT was not detected by week nine, while, from week 20 onwards, only the β-splice version was expressed [9]. Thus, a downregulation of TA correlated best with the disappearance of the WT hTERT without any direct evidence of splice product expression being involved.

In contrast, in the fetal heart, TA, WT and α-spliced hTERT were all simultaneously downregulated by gestational week 13/pcw 11 [9]. In liver, however, TA was maintained over development with WT TERT and α-spliced hTERT being present [9]. Maintenance of TA directly correlated with telomere maintenance in liver, while in heart and kidney it decreased after TA was downregulated [9]. TA was also maintained in other tissues until gestational weeks 21 (19 pcw) of fetal development in lung, spleen, and testis while it was lost earlier in heart, brain and kidney [8]. This is most likely due to the fact that human adult liver tissue still possesses TA [50], adult spleen contains telomerase positive lymphocytes while in lung tissue from adults no TA was found [51].

### NSPCs

Neural stem cells emerge very early during embryonic development and produce all of the different cells of the brain and central nervous system [40]. In order to establish how much neural precursor stem cells (NPSCs) contributed to TA in the development of early gestational brains, we used primary NPSCs from forebrain derived at 6 pcw. It is well known that NPSCs differentiate into various cell types of the brain, predominantly neurons and astrocytes with neurogenesis preceding astrogenesis [41,42]. *In vitro* differentiation of NPSCs usually generates a mix of neurons (70%) and astrocytes (30%) after two weeks [36]. During the differentiation, TA decreased late in differentiation, after 6 days. Surprisingly, TA was higher in the brain tissue from 6 pcw compared to the NPSCs derived from a similar time point. This was most likely caused by an extensive *in vitro* expansion of these NPSCs during their *in vitro* cultivation via neurospheres prior to their differentiation [35], exhausting some of the stem and progenitor cell potential including telomerase activity. However, the TA was comparable to that shown by Zhang *et al.* in primary NPSCs derived from gestational week 12 [52].

We did not find any changes in the expression of telomerase-related genes that could explain the decrease in telomerase activity during the *in vitro* differentiation process of hNPSCs. This result suggests that brain development *in vivo* might involve different mechanisms of downregulation of telomerase activity than in NPSCs during *in vitro* differentiation. However, the downregulation of TA *in vitro* in human NPSCS is comparable to that found during *in vitro* culture of mouse neuronal cells from embryonic day 15 that declined within the same time frame (after day eight and undetectable at day 14) [3]. However, the exact mechanisms for this downregulation of TA remain elusive.

It is known that a decrease in telomerase activity correlates to differentiation of human embryonic stem cells (hESCs) [53]. We demonstrated previously that in spontaneous differentiation of hESCs over 20 days telomerase activity is downregulated towards the end of the differentiation period. This is preceded by a strong decrease in the expression of both telomerase genes after day seven of differentiation, and might be partly due to promoter modifications for hTERT and hTR [53]. This kinetics was different from the one we show here in NPSCs where TA is downregulated without changes in the analyzed telomerase-related genes. Radan and colleagues showed that hTERT splicing pattern rapidly induced spontaneous hESC differentiation emphasizing the importance of specific ratios of different hTERT splice products for high telomerase activity that is necessary for indefinite proliferation of human ESCs [12].

In general, telomerase activity is regulated by many different endogenous and exogenous factors (for review see [54]). Downregulation of telomerase activity during differentiation of stem cells is rather well documented [55], however *in vitro* differentiation of NPSCs did not model the expression kinetics of telomerase-related genes during downregulation of telomerase activity.

## 5. Conclusions

In conclusion, the main finding of our study is that telomerase activity is downregulated between pcw 6 and 14/17 during human fetal brain development. Our data suggest that a strong decrease in hTR expression might causally contribute to the downregulation of telomerase activity. In addition, both hTERT variants (wild type and α-spliced hTERT) seem to co-exist in neurons. It would be interesting to determine whether α-spliced hTERT is able to exert a protective function similar to those demonstrated for other hTERT splice versions previously [6,16].

Furthermore, our results also suggest that downregulation of TA in NPSCs *in vitro* does not seem to be regulated by the same mechanisms as during brain development *in vivo*.

## Figures and Tables

**Table 1:** Description of brain tissues used.

Age	Age group	Source
Carnegie Stage 17	6 pcw	HDBR
Carnegie Stage 17	6 pcw	HDBR
Carnegie Stage 17	6 pcw	HDBR
10 pcw	10–17 pcw	HDBR
10 pcw	10–17 pcw	HDBR
14 pcw	10–17 pcw	HDBR
14 pcw	10–17 pcw	HDBR
17 pcw	10–17 pcw	HDBR
17 pcw	10–17 pcw	HDBR
19 pcw	19–20 pcw	HDBR
19 pcw	19–20 pcw	HDBR
20 pcw	19–20 pcw	HDBR
15 years old	Young	NBTR
20 years old	Young	NBTR
20 years old	Young	NBTR
68 years old	Old	NBTR
73 years old	Old	NBTR
78 years old	Old	NBTR

HDBR = Human Developmental Biology Resource; NBTR = Newcastle Brain Tissue Resource; PCW = post-conception weeks; Gestational weeks (GW) are 2 weeks less that than pcw.

**Table 2:** Primers used in qPCR. * Two annealing temperatures are provided for the hTERT reverse primer since the temperature was dependent on the respective forward primer used (WT or α hTERT).

Primer	Sequence	Annealing Temperature (°C)	Ref.
hGAPDH fw	TGCACCACCAACTGCTTAGC	60	[37]
hGAPDH rev	GGCATGGACTGTGGTCATGA	60	[37]
hTR fw	GCCTTCCACCGTTCATTCTA	60	[38]
hTR rev	CCTGAAAGGCCTGAACCTC	60	[38]
WT hTERT fw	TGTACTTTGTCAAGGTGGATGTG	60	[14]
α-hTERT fw	CTGAGCTGTACTTTGTCAAGGAC	53	[15]
hTERT rev	GTACGGCTGGAGGTCTGTCAA	60/53 *	[14,15]

**Table 3:** Comparison of telomerase activity and expression of telomerase-related genes. Expression of hTR, WT hTERT and α-spliced hTERT in fetal heart, kidney and liver refer to data from [8,9] while data for brain are from this study. Numbers correspond to post-conception weeks at which hTR, WT hTERT or α-spliced hTERT were downregulated. PCW is two weeks more than the gestational weeks shown in [8,9].

Tissue	Decrease of TA (PCW)	hTR expression (PCW)	WT hTERT expression (PCW)	α-spliced hTERT expression (PCW)
Heart	11	maintained	11	Not detected
Kidney	15	maintained	16	9
Liver, Lung, Spleen	maintained	maintained	maintained	maintained
Brain	17	Downregulated from 6 towards 19/20	19/20	Increased from 6 towards 19/20

## Abbreviations

The following abbreviations are used in this manuscript:

GW	gestational week
h	human
NPSC	neural precursor stem cells
pcw	post conception weeks
ROS	reactive oxygen species
TA	telomerase activity
TR	RNA component of telomerase
TERT	telomerase reverse transcriptase
WT	wild type
